# {2,7-Dimeth­oxy-8-[4-(2-methyl­prop­yl)benzo­yl]naphthalen-1-yl}[4-(2-methyl­prop­yl)phen­yl]methanone

**DOI:** 10.1107/S1600536812045953

**Published:** 2012-11-14

**Authors:** Kosuke Sasagawa, Daichi Hijikata, Rei Sakamoto, Akiko Okamoto, Noriyuki Yonezawa

**Affiliations:** a Department of Organic and Polymer Materials Chemistry, Tokyo University of Agriculture & Technology, Koganei, Tokyo 184-8588, Japan

## Abstract

In the mol­ecule of the title compound, C_34_H_36_O_4_, the two 4-isobutyl­benzoyl groups at the 1- and 8-positions of the naphthalene ring system are aligned almost anti­parallel, and the benzene rings make a dihedral angle of 21.59 (7)°. The dihedral angles between the benzene rings and the naphthalene ring system are 69.26 (6) and 64.29 (5)°. There are no classical hydrogen bonds in the structure, but inversion-related mol­ecules engage in π–π stacking, with an inter­planar spacing between related naphthalene groups of 3.4120 (16) Å.

## Related literature

For details of the formation reaction of aroylated naphthalene compounds *via* electrophilic aromatic substitution of naphthalene derivatives, see: Okamoto & Yonezawa (2009 ▶); Okamoto *et al.* (2011 ▶). For the structures of closely related compounds, see: Hijikata *et al.* (2010 ▶); Muto *et al.* (2010 ▶); Sasagawa, Hijikata *et al.* (2011 ▶); Sasagawa, Muto *et al.* (2011 ▶); Sasagawa *et al.* (2012 ▶).
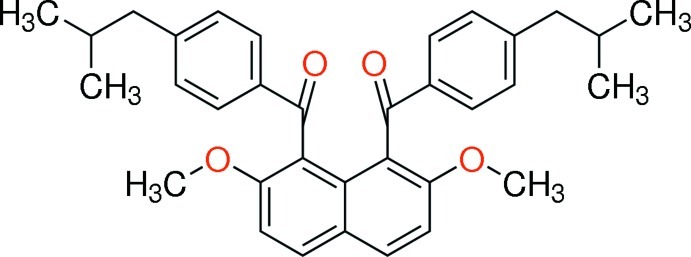


## Experimental

### 

#### Crystal data

C_34_H_36_O_4_
*M* *_r_* = 508.63
Monoclinic, 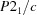

*a* = 18.5280 (4) Å
*b* = 7.83885 (15) Å
*c* = 20.2304 (4) Å
β = 103.642 (1)°
*V* = 2855.33 (10) Å^3^
*Z* = 4
Cu *K*α radiation
μ = 0.60 mm^−1^
*T* = 193 K
0.60 × 0.40 × 0.05 mm

#### Data collection

Rigaku R-AXIS RAPID diffractometer
Absorption correction: numerical (*NUMABS*; Higashi, 1999 ▶) *T*_min_ = 0.714, *T*_max_ = 0.971
50913 measured reflections
5237 independent reflections
3838 reflections with *I* > 2σ(*I*)
*R*_int_ = 0.052

#### Refinement

*R*[*F* ^2^ > 2σ(*F* ^2^)] = 0.042
*wR*(*F* ^2^) = 0.126
*S* = 1.12
5237 reflections
350 parameters
H-atom parameters constrained
Δρ_max_ = 0.17 e Å^−3^
Δρ_min_ = −0.15 e Å^−3^

### 

Data collection: *PROCESS-AUTO* (Rigaku, 1998 ▶); cell refinement: *PROCESS-AUTO*; data reduction: *PROCESS-AUTO*; program(s) used to solve structure: *SIR2004* (Burla *et al.*, 2005 ▶); program(s) used to refine structure: *SHELXL97* (Sheldrick, 2008 ▶); molecular graphics: *ORTEPIII* (Burnett & Johnson, 1996 ▶); software used to prepare material for publication: *SHELXL97*.

## Supplementary Material

Crystal structure: contains datablock(s) I, global. DOI: 10.1107/S1600536812045953/pk2455sup1.cif

Structure factors: contains datablock(s) I. DOI: 10.1107/S1600536812045953/pk2455Isup2.hkl

Supplementary material file. DOI: 10.1107/S1600536812045953/pk2455Isup3.cml

Additional supplementary materials:  crystallographic information; 3D view; checkCIF report

## Figures and Tables

**Fig. 1.**
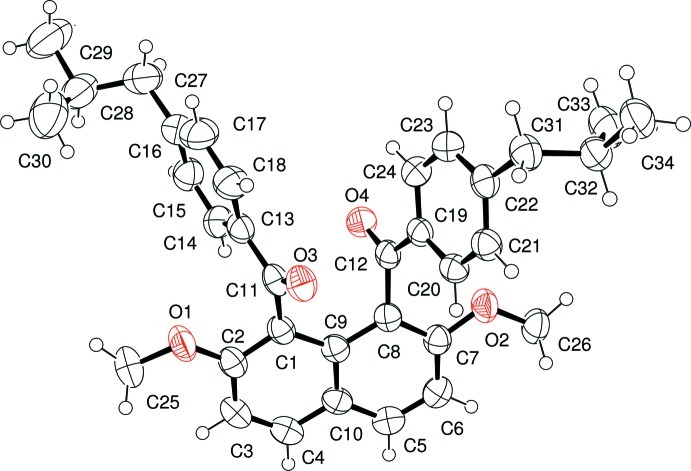
An ellipsoid plot of the title compound (50% probability). Hydrogen atoms are shown as small spheres of arbitrary radius.

**Fig. 2.**
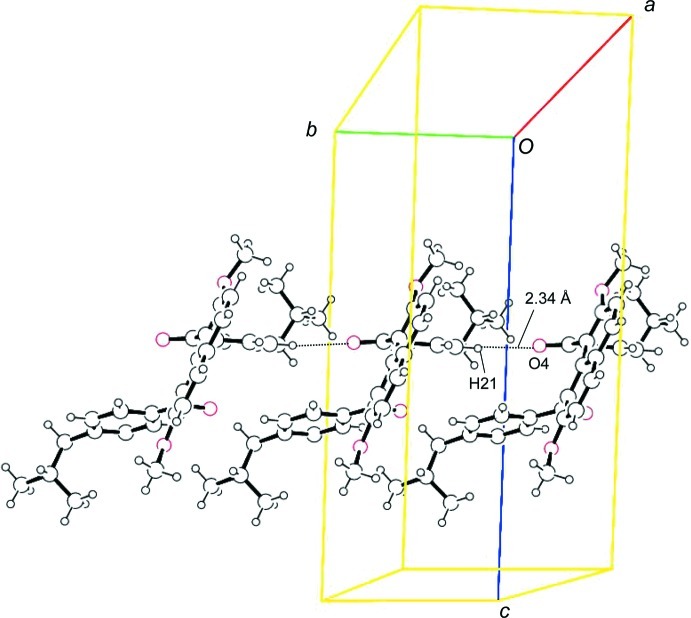
A view of the packing of the title compound viewed roughly down the crystallographic a axis. A chain of C—H···O interactions that propagates parallel to b is indicated by dotted lines.

## supplementary crystallographic information

### Comment

In the course of our study on selective electrophilic aromatic aroylation of the naphthalene ring core, 1,8-diaroylnaphthalene compounds have proved to be formed regioselectively by the aid of a suitable acidic mediator (Okamoto & Yonezawa, 2009, Okamoto *et al.*, 2011). Recently, we have reported the X-ray crystal structures of 1,8-diaroylated 2,7-dimethoxynaphthalene derivatives such as [2,7-dimethoxy-8-(4-methylbenzoyl)-1-naphthyl](4-methylphenyl)methanone [1,8-bis(4-methylbenzoyl)-2,7-dimethoxynaphthalene] (Muto *et al.*, 2010), {8-[4-(bromomethyl)benzoyl]-2,7-dimethoxynaphthalen-1-yl}[4-(bromomethyl)phenyl]methanone [1,8-bis(4-bromomethylbenzoyl)-2,7-dimethoxynaphthalene] (Sasagawa, Hijikata *et al.*, 2011), {8-[4-(butoxy)benzoyl]-2,7-dimethoxynaphthalen-1-yl}[4-(butoxy)phenyl]methanone [1,8-bis(4-butoxylbenzoyl)-2,7-dimethoxynaphthalene] (Sasagawa, Muto *et al.*, 2011), [2,7-dimethoxy-8-(4-propylbenzoyl)-naphthalen-1-yl](4-propylphenyl)-methanone [1,8-bis(4-butoxylbenzoyl)-2,7-dimethoxynaphthalene] (Sasagawa *et al.*, 2012). The aroyl groups in these compounds are almost perpendicular to the naphthalene rings, and are oriented in opposite directions (*anti*-orientation). Moreover, we have also clarified that the aroyl groups of 2,7-dimethoxy-1,8-bis(4-phenoxybenzoyl)naphthalene (Hijikata *et al.*, 2010) are oriented in the same direction (*syn*-orientation). As part of our ongoing studies on the molecular structures of these kinds of homologous molecules, the X-ray crystal structure of the title compound, 1,8-diaroylatednaphthalene bearing isobutyl groups, is discussed in this article.

The molecular structure of the title compound is displayed in Fig 1. Two 4-isobutylbenzoyl groups are situated in the *anti*-orientation. The dihedral angle between the best planes of the two phenyl rings is 21.59 (7)°. The dihedral angles between the best planes of the 4-isobutylphenyl rings and the naphthalene ring are 69.26 (6)° and 64.29 (5)°.

The C═O bond of the ketonic carbonyl moiety (C12═O4), carbon atom (C31) of isobutyl groups, and benzene ring lie on the same plane [torsion angles O4—C12—C19—C24 = 2.49 (18)°; C31—C22—C23—C24 = 176.11 (14)°]. The corresponding torsion angles in the other aroyl group are 172.86 (12)° [O3—C11—C13—C14] and 178.36 (17)° [C27—C16—C17—C18], respectively.

In the molecular packing, C—H···O interactions between the carbonyl oxygen atoms and hydrogen atoms of benzene ring are observed along *b* axis. The C—H···O interactions effectively contribute to stabilization of the molecular alignment (C21—H21···O4 = 2.34 Å; symmetry code: *x*,-1 + *y*, *z*; Fig. 2).

### Experimental

To a 50 ml flask, 4-isobutylbenzoic acid (1.96 g, 11.0 mmol), phosphorus pentoxide–methanesulfonic acid mixture (P_2_O_5_–MsOH [1/10 *w*/*w*]; 22.0 ml) were placed and stirred at 333 K. To the solution thus obtained, 2,7-dimethoxynaphthalene (941 mg, 5.0 mmol) was added. After the reaction mixture was stirred at 333 K for 1.0 h, the reaction mixture was poured into ice-cold water (30 ml). The aqueous layer was extracted with CHCl_3_ (15 ml × 3). The combined extracts were washed with 2 *M* aqueous NaOH followed by washing with brine. The organic layers thus obtained were dried over anhydrous MgSO_4_. The solvent was removed under reduced pressure to give cake (100% yield). The crude product was purified by recrystallization from methanol (32% yield). Colorless platelet single crystals suitable for X-ray diffraction were obtained by repeated crystallization from ethanol.

Spectroscopic Data:

^1^H-NMR δ (300 MHz, CDCl_3_): 0.91 (12*H*, d, *J* = 6.6 Hz), 1.89 (2*H*, m, *J*= 6.6 Hz), 2.49 (4*H*, d, *J* = 6.6 Hz), 3.68 (6*H*, s), 7.09 (4*H*, d, *J* = 7.5 Hz), 7.20 (2*H*, d, *J* = 9.0 Hz), 7.59(2*H*, d, *J* = 7.5 Hz), 7.95 (4*H*, d, *J* = 9.0 Hz)

^13^C-NMR δ (75 MHz, CDCl_3_): 22.3, 29.3, 45.4, 56.2, 111.1, 121.5, 125.3, 128.5, 128.9, 129.4, 131.7, 136.4, 146.7, 156.0, 196.2 p.p.m.

IR (KBr): 2952 (CH_3_), 2911 (CH_2_), 1652 (C=O), 1605, 1510, 1460 (Ar) cm^-1^

HRMS (m/*z*): [*M*+H]^+^ calcd. for C_34_H_37_O_4_, 509.2692, found, 509.2608

m.p.= 472.0—474.0 K

### Refinement

All H atoms were found in a difference map and were subsequently refined as riding atoms, with C—H = 0.95 (aromatic), 0.98 (methyl) Å, 0.99 (methylene) and 1.00 (methyne) with *U*_ĩso_(H) = 1.2 *U*_eq_(C).

### Crystal data

**Table:**

C_34_H_36_O_4_	*F*(000) = 1088
*M**_r_* = 508.63	*D*_x_ = 1.183 Mg m^−^^3^
Monoclinic, *P*2_1_/*c*	Cu *K*α radiation, λ = 1.54187 Å
Hall symbol: -P 2ybc	Cell parameters from 26694 reflections
*a* = 18.5280 (4) Å	θ = 3.7–68.3°
*b* = 7.83885 (15) Å	µ = 0.60 mm^−^^1^
*c* = 20.2304 (4) Å	*T* = 193 K
β = 103.642 (1)°	Platelet, colorless
*V* = 2855.33 (10) Å^3^	0.60 × 0.40 × 0.05 mm
*Z* = 4	

### Data collection

**Table:**

Rigaku R-AXIS RAPID diffractometer	5237 independent reflections
Radiation source: fine-focus sealed tube	3838 reflections with *I* > 2σ(*I*)
Graphite monochromator	*R*_int_ = 0.052
ω scans	θ_max_ = 68.2°, θ_min_ = 4.5°
Absorption correction: numerical (*NUMABS*; Higashi, 1999)	*h* = −21→22
*T*_min_ = 0.714, *T*_max_ = 0.971	*k* = −9→9
50913 measured reflections	*l* = −24→24

### Refinement

**Table:**

Refinement on *F*^2^	Secondary atom site location: difference Fourier map
Least-squares matrix: full	Hydrogen site location: inferred from neighbouring sites
*R*[*F*^2^ > 2σ(*F*^2^)] = 0.042	H-atom parameters constrained
*wR*(*F*^2^) = 0.126	*w* = 1/[σ^2^(*F*_o_^2^) + (0.0662*P*)^2^] where *P* = (*F*_o_^2^ + 2*F*_c_^2^)/3
*S* = 1.12	(Δ/σ)_max_ < 0.001
5237 reflections	Δρ_max_ = 0.17 e Å^−^^3^
350 parameters	Δρ_min_ = −0.15 e Å^−^^3^
0 restraints	Extinction correction: *SHELXL97* (Sheldrick, 2008), Fc^*^=kFc[1+0.001xFc^2^λ^3^/sin(2θ)]^-1/4^
Primary atom site location: structure-invariant direct methods	Extinction coefficient: 0.0022 (2)

### Special details

**Table:**

Geometry. All e.s.d.'s (except the e.s.d. in the dihedral angle between two l.s. planes) are estimated using the full covariance matrix. The cell e.s.d.'s are taken into account individually in the estimation of e.s.d.'s in distances, angles and torsion angles; correlations between e.s.d.'s in cell parameters are only used when they are defined by crystal symmetry. An approximate (isotropic) treatment of cell e.s.d.'s is used for estimating e.s.d.'s involving l.s. planes.
Refinement. Refinement of *F*^2^ against ALL reflections. The weighted *R*-factor *wR* and goodness of fit *S* are based on *F*^2^, conventional *R*-factors *R* are based on *F*, with *F* set to zero for negative *F*^2^. The threshold expression of *F*^2^ > σ(*F*^2^) is used only for calculating *R*-factors(gt) *etc*. and is not relevant to the choice of reflections for refinement. *R*-factors based on *F*^2^ are statistically about twice as large as those based on *F*, and *R*- factors based on ALL data will be even larger.

### Fractional atomic coordinates and isotropic or equivalent isotropic displacement parameters (Å^2^)

**Table:**

	*x*	*y*	*z*	*U*_iso_*/*U*_eq_	
O1	0.66145 (6)	0.32112 (15)	0.61940 (5)	0.0635 (3)	
O2	0.53292 (5)	0.00068 (13)	0.27977 (5)	0.0576 (3)	
O3	0.71658 (5)	0.02470 (13)	0.51300 (5)	0.0553 (3)	
O4	0.67422 (5)	0.26567 (12)	0.36980 (5)	0.0527 (3)	
C1	0.62041 (8)	0.22224 (18)	0.50872 (7)	0.0453 (3)	
C2	0.60247 (8)	0.29086 (19)	0.56568 (7)	0.0509 (4)	
C3	0.52803 (9)	0.3152 (2)	0.56905 (8)	0.0565 (4)	
H3	0.5168	0.3669	0.6079	0.068*	
C4	0.47275 (9)	0.26432 (19)	0.51642 (8)	0.0555 (4)	
H4	0.4227	0.2778	0.5195	0.067*	
C5	0.42902 (8)	0.13689 (19)	0.40346 (8)	0.0537 (4)	
H5	0.3793	0.1477	0.4078	0.064*	
C6	0.44203 (8)	0.0687 (2)	0.34550 (8)	0.0547 (4)	
H6	0.4019	0.0305	0.3102	0.066*	
C7	0.51556 (8)	0.05545 (18)	0.33848 (7)	0.0478 (4)	
C8	0.57515 (7)	0.10547 (17)	0.38983 (6)	0.0434 (3)	
C9	0.56242 (7)	0.17307 (17)	0.45187 (6)	0.0440 (3)	
C10	0.48732 (8)	0.19171 (18)	0.45706 (7)	0.0478 (4)	
C11	0.70027 (8)	0.1766 (2)	0.51402 (6)	0.0469 (4)	
C12	0.65026 (7)	0.12146 (18)	0.37335 (6)	0.0428 (3)	
C13	0.75748 (8)	0.3109 (2)	0.52059 (6)	0.0481 (4)	
C14	0.73950 (9)	0.4833 (2)	0.51447 (7)	0.0545 (4)	
H14	0.6890	0.5170	0.5068	0.065*	
C15	0.79377 (9)	0.6062 (2)	0.51933 (8)	0.0600 (4)	
H15	0.7802	0.7233	0.5149	0.072*	
C16	0.86786 (9)	0.5603 (2)	0.53064 (8)	0.0638 (4)	
C17	0.88575 (9)	0.3888 (3)	0.53645 (10)	0.0737 (5)	
H17	0.9363	0.3555	0.5441	0.088*	
C18	0.83170 (9)	0.2650 (2)	0.53138 (8)	0.0641 (4)	
H18	0.8454	0.1479	0.5353	0.077*	
C19	0.69369 (8)	−0.02869 (17)	0.36131 (6)	0.0422 (3)	
C20	0.66833 (8)	−0.19549 (18)	0.36293 (6)	0.0465 (4)	
H20	0.6214	−0.2158	0.3729	0.056*	
C21	0.71025 (8)	−0.33134 (19)	0.35037 (7)	0.0498 (4)	
H21	0.6919	−0.4441	0.3518	0.060*	
C22	0.77954 (8)	−0.30609 (19)	0.33551 (7)	0.0486 (4)	
C23	0.80510 (8)	−0.1394 (2)	0.33565 (8)	0.0550 (4)	
H23	0.8524	−0.1189	0.3267	0.066*	
C24	0.76339 (8)	−0.00324 (19)	0.34854 (7)	0.0515 (4)	
H24	0.7825	0.1093	0.3487	0.062*	
C25	0.65039 (10)	0.4214 (2)	0.67461 (8)	0.0731 (5)	
H25A	0.6249	0.5275	0.6572	0.088*	
H25B	0.6986	0.4483	0.7050	0.088*	
H25C	0.6200	0.3577	0.6998	0.088*	
C26	0.47399 (9)	−0.0512 (2)	0.22480 (7)	0.0619 (4)	
H26A	0.4946	−0.0948	0.1877	0.074*	
H26B	0.4417	0.0466	0.2086	0.074*	
H26C	0.4451	−0.1412	0.2402	0.074*	
C27	0.92724 (10)	0.6961 (3)	0.53805 (11)	0.0823 (6)	
H27A	0.9138	0.7750	0.4989	0.099*	
H27B	0.9748	0.6408	0.5364	0.099*	
C28	0.93864 (10)	0.7998 (3)	0.60370 (11)	0.0817 (6)	
H28	0.8907	0.8588	0.6035	0.098*	
C29	0.99748 (11)	0.9370 (3)	0.60563 (15)	0.1205 (9)	
H29A	1.0451	0.8829	0.6053	0.145*	
H29B	1.0028	1.0053	0.6471	0.145*	
H29C	0.9824	1.0111	0.5657	0.145*	
C30	0.95729 (12)	0.6876 (3)	0.66594 (12)	0.1083 (8)	
H30A	0.9650	0.7586	0.7069	0.130*	
H30B	1.0027	0.6232	0.6663	0.130*	
H30C	0.9163	0.6081	0.6652	0.130*	
C31	0.82253 (8)	−0.4543 (2)	0.31722 (7)	0.0565 (4)	
H31A	0.8271	−0.5429	0.3528	0.068*	
H31B	0.8732	−0.4154	0.3168	0.068*	
C32	0.78695 (9)	−0.5339 (2)	0.24818 (8)	0.0579 (4)	
H32	0.7387	−0.5866	0.2514	0.069*	
C33	0.77059 (10)	−0.4010 (3)	0.19229 (8)	0.0755 (5)	
H33A	0.7348	−0.3180	0.2019	0.091*	
H33B	0.7497	−0.4567	0.1486	0.091*	
H33C	0.8167	−0.3422	0.1902	0.091*	
C34	0.83574 (9)	−0.6740 (2)	0.23036 (9)	0.0711 (5)	
H34A	0.8819	−0.6240	0.2235	0.085*	
H34B	0.8095	−0.7311	0.1885	0.085*	
H34C	0.8473	−0.7571	0.2675	0.085*	

### Atomic displacement parameters (Å^2^)

**Table:**

	*U*^11^	*U*^22^	*U*^33^	*U*^12^	*U*^13^	*U*^23^
O1	0.0687 (7)	0.0842 (8)	0.0372 (5)	0.0057 (6)	0.0118 (5)	−0.0101 (5)
O2	0.0578 (6)	0.0690 (8)	0.0419 (5)	−0.0008 (5)	0.0037 (4)	−0.0095 (5)
O3	0.0656 (7)	0.0522 (7)	0.0466 (6)	0.0076 (5)	0.0103 (5)	0.0012 (5)
O4	0.0626 (6)	0.0452 (6)	0.0515 (6)	−0.0059 (5)	0.0161 (5)	0.0028 (5)
C1	0.0543 (8)	0.0444 (9)	0.0385 (7)	0.0016 (6)	0.0132 (6)	0.0038 (6)
C2	0.0615 (9)	0.0520 (9)	0.0402 (7)	0.0029 (7)	0.0144 (7)	0.0031 (6)
C3	0.0670 (10)	0.0617 (10)	0.0456 (8)	0.0080 (8)	0.0229 (7)	0.0026 (7)
C4	0.0595 (9)	0.0571 (10)	0.0549 (9)	0.0059 (7)	0.0238 (7)	0.0094 (7)
C5	0.0483 (8)	0.0528 (10)	0.0607 (9)	−0.0002 (7)	0.0139 (7)	0.0073 (7)
C6	0.0525 (9)	0.0526 (10)	0.0552 (9)	−0.0028 (7)	0.0049 (7)	−0.0003 (7)
C7	0.0523 (8)	0.0459 (9)	0.0436 (8)	−0.0006 (7)	0.0082 (6)	0.0009 (6)
C8	0.0505 (8)	0.0394 (8)	0.0399 (7)	0.0011 (6)	0.0098 (6)	0.0043 (6)
C9	0.0520 (8)	0.0402 (8)	0.0405 (7)	0.0011 (6)	0.0120 (6)	0.0064 (6)
C10	0.0525 (8)	0.0447 (9)	0.0484 (8)	0.0018 (7)	0.0165 (7)	0.0079 (6)
C11	0.0587 (9)	0.0518 (10)	0.0294 (6)	0.0052 (7)	0.0090 (6)	0.0023 (6)
C12	0.0529 (8)	0.0437 (9)	0.0304 (6)	−0.0037 (7)	0.0072 (6)	0.0026 (6)
C13	0.0525 (9)	0.0558 (10)	0.0358 (7)	0.0014 (7)	0.0099 (6)	−0.0001 (6)
C14	0.0521 (9)	0.0589 (11)	0.0492 (8)	0.0019 (7)	0.0056 (7)	0.0025 (7)
C15	0.0640 (10)	0.0599 (11)	0.0540 (9)	−0.0040 (8)	0.0094 (7)	0.0004 (8)
C16	0.0617 (10)	0.0731 (12)	0.0600 (10)	−0.0103 (9)	0.0213 (8)	−0.0056 (8)
C17	0.0517 (10)	0.0813 (14)	0.0927 (13)	0.0007 (9)	0.0260 (9)	−0.0087 (11)
C18	0.0587 (10)	0.0632 (11)	0.0731 (11)	0.0066 (8)	0.0207 (8)	−0.0032 (9)
C19	0.0499 (8)	0.0452 (9)	0.0311 (6)	−0.0029 (6)	0.0088 (6)	0.0016 (6)
C20	0.0533 (8)	0.0480 (9)	0.0401 (7)	−0.0033 (7)	0.0149 (6)	0.0020 (6)
C21	0.0593 (9)	0.0467 (9)	0.0450 (8)	−0.0014 (7)	0.0157 (7)	0.0011 (6)
C22	0.0532 (9)	0.0534 (10)	0.0366 (7)	0.0029 (7)	0.0055 (6)	−0.0010 (6)
C23	0.0484 (8)	0.0610 (10)	0.0569 (9)	−0.0039 (7)	0.0155 (7)	−0.0073 (7)
C24	0.0524 (9)	0.0503 (9)	0.0523 (8)	−0.0067 (7)	0.0134 (7)	−0.0032 (7)
C25	0.0917 (13)	0.0783 (12)	0.0477 (9)	0.0079 (10)	0.0132 (8)	−0.0148 (9)
C26	0.0693 (10)	0.0641 (11)	0.0460 (8)	−0.0106 (8)	0.0011 (7)	−0.0051 (7)
C27	0.0692 (12)	0.0886 (15)	0.0965 (14)	−0.0146 (10)	0.0342 (10)	−0.0076 (11)
C28	0.0573 (11)	0.0841 (14)	0.1007 (15)	−0.0095 (10)	0.0126 (10)	−0.0137 (12)
C29	0.0759 (14)	0.1112 (19)	0.173 (3)	−0.0314 (13)	0.0276 (15)	−0.0368 (18)
C30	0.0873 (15)	0.125 (2)	0.0978 (17)	0.0013 (14)	−0.0075 (12)	−0.0127 (15)
C31	0.0556 (9)	0.0624 (10)	0.0497 (8)	0.0084 (8)	0.0089 (7)	−0.0037 (7)
C32	0.0522 (9)	0.0668 (11)	0.0553 (9)	0.0009 (8)	0.0141 (7)	−0.0109 (8)
C33	0.0761 (12)	0.1001 (15)	0.0466 (9)	0.0150 (11)	0.0072 (8)	−0.0064 (9)
C34	0.0699 (11)	0.0795 (13)	0.0663 (11)	0.0052 (9)	0.0208 (9)	−0.0192 (9)

### Geometric parameters (Å, º)

**Table:**

O1—C2	1.3679 (17)	C20—H20	0.9500
O1—C25	1.4198 (17)	C21—C22	1.4000 (19)
O2—C7	1.3707 (16)	C21—H21	0.9500
O2—C26	1.4218 (16)	C22—C23	1.389 (2)
O3—C11	1.2297 (16)	C22—C31	1.5034 (19)
O4—C12	1.2227 (15)	C23—C24	1.3786 (19)
C1—C2	1.3814 (18)	C23—H23	0.9500
C1—C9	1.4292 (19)	C24—H24	0.9500
C1—C11	1.502 (2)	C25—H25A	0.9800
C2—C3	1.410 (2)	C25—H25B	0.9800
C3—C4	1.352 (2)	C25—H25C	0.9800
C3—H3	0.9500	C26—H26A	0.9800
C4—C10	1.4113 (19)	C26—H26B	0.9800
C4—H4	0.9500	C26—H26C	0.9800
C5—C6	1.361 (2)	C27—C28	1.528 (3)
C5—C10	1.4053 (19)	C27—H27A	0.9900
C5—H5	0.9500	C27—H27B	0.9900
C6—C7	1.406 (2)	C28—C30	1.508 (3)
C6—H6	0.9500	C28—C29	1.526 (3)
C7—C8	1.3823 (18)	C28—H28	1.0000
C8—C9	1.4322 (18)	C29—H29A	0.9800
C8—C12	1.5107 (18)	C29—H29B	0.9800
C9—C10	1.4274 (18)	C29—H29C	0.9800
C11—C13	1.478 (2)	C30—H30A	0.9800
C12—C19	1.4781 (18)	C30—H30B	0.9800
C13—C18	1.388 (2)	C30—H30C	0.9800
C13—C14	1.391 (2)	C31—C32	1.530 (2)
C14—C15	1.379 (2)	C31—H31A	0.9900
C14—H14	0.9500	C31—H31B	0.9900
C15—C16	1.385 (2)	C32—C33	1.515 (2)
C15—H15	0.9500	C32—C34	1.519 (2)
C16—C17	1.383 (3)	C32—H32	1.0000
C16—C27	1.513 (2)	C33—H33A	0.9800
C17—C18	1.381 (2)	C33—H33B	0.9800
C17—H17	0.9500	C33—H33C	0.9800
C18—H18	0.9500	C34—H34A	0.9800
C19—C24	1.3898 (19)	C34—H34B	0.9800
C19—C20	1.3923 (18)	C34—H34C	0.9800
C20—C21	1.3765 (18)		
			
C2—O1—C25	118.99 (12)	C21—C22—C31	120.63 (13)
C7—O2—C26	118.26 (11)	C24—C23—C22	121.51 (14)
C2—C1—C9	119.55 (13)	C24—C23—H23	119.2
C2—C1—C11	117.40 (12)	C22—C23—H23	119.2
C9—C1—C11	122.30 (12)	C23—C24—C19	120.72 (14)
O1—C2—C1	115.22 (13)	C23—C24—H24	119.6
O1—C2—C3	123.04 (13)	C19—C24—H24	119.6
C1—C2—C3	121.58 (13)	O1—C25—H25A	109.5
C4—C3—C2	119.34 (14)	O1—C25—H25B	109.5
C4—C3—H3	120.3	H25A—C25—H25B	109.5
C2—C3—H3	120.3	O1—C25—H25C	109.5
C3—C4—C10	121.85 (14)	H25A—C25—H25C	109.5
C3—C4—H4	119.1	H25B—C25—H25C	109.5
C10—C4—H4	119.1	O2—C26—H26A	109.5
C6—C5—C10	121.66 (13)	O2—C26—H26B	109.5
C6—C5—H5	119.2	H26A—C26—H26B	109.5
C10—C5—H5	119.2	O2—C26—H26C	109.5
C5—C6—C7	119.25 (14)	H26A—C26—H26C	109.5
C5—C6—H6	120.4	H26B—C26—H26C	109.5
C7—C6—H6	120.4	C16—C27—C28	114.11 (15)
O2—C7—C8	115.53 (12)	C16—C27—H27A	108.7
O2—C7—C6	122.79 (13)	C28—C27—H27A	108.7
C8—C7—C6	121.61 (13)	C16—C27—H27B	108.7
C7—C8—C9	119.74 (12)	C28—C27—H27B	108.7
C7—C8—C12	118.15 (12)	H27A—C27—H27B	107.6
C9—C8—C12	120.88 (12)	C30—C28—C29	111.62 (19)
C10—C9—C1	118.29 (12)	C30—C28—C27	111.91 (18)
C10—C9—C8	117.86 (12)	C29—C28—C27	110.40 (18)
C1—C9—C8	123.85 (12)	C30—C28—H28	107.6
C5—C10—C4	120.89 (13)	C29—C28—H28	107.6
C5—C10—C9	119.80 (13)	C27—C28—H28	107.6
C4—C10—C9	119.30 (13)	C28—C29—H29A	109.5
O3—C11—C13	121.17 (13)	C28—C29—H29B	109.5
O3—C11—C1	118.13 (14)	H29A—C29—H29B	109.5
C13—C11—C1	120.70 (13)	C28—C29—H29C	109.5
O4—C12—C19	120.49 (12)	H29A—C29—H29C	109.5
O4—C12—C8	117.11 (12)	H29B—C29—H29C	109.5
C19—C12—C8	122.40 (12)	C28—C30—H30A	109.5
C18—C13—C14	118.28 (15)	C28—C30—H30B	109.5
C18—C13—C11	119.50 (14)	H30A—C30—H30B	109.5
C14—C13—C11	122.19 (13)	C28—C30—H30C	109.5
C15—C14—C13	121.14 (15)	H30A—C30—H30C	109.5
C15—C14—H14	119.4	H30B—C30—H30C	109.5
C13—C14—H14	119.4	C22—C31—C32	113.63 (12)
C14—C15—C16	120.51 (16)	C22—C31—H31A	108.8
C14—C15—H15	119.7	C32—C31—H31A	108.8
C16—C15—H15	119.7	C22—C31—H31B	108.8
C17—C16—C15	118.37 (16)	C32—C31—H31B	108.8
C17—C16—C27	121.43 (16)	H31A—C31—H31B	107.7
C15—C16—C27	120.19 (17)	C33—C32—C34	110.21 (13)
C18—C17—C16	121.46 (16)	C33—C32—C31	111.58 (14)
C18—C17—H17	119.3	C34—C32—C31	111.05 (13)
C16—C17—H17	119.3	C33—C32—H32	107.9
C17—C18—C13	120.23 (16)	C34—C32—H32	107.9
C17—C18—H18	119.9	C31—C32—H32	107.9
C13—C18—H18	119.9	C32—C33—H33A	109.5
C24—C19—C20	118.21 (13)	C32—C33—H33B	109.5
C24—C19—C12	118.82 (12)	H33A—C33—H33B	109.5
C20—C19—C12	122.96 (12)	C32—C33—H33C	109.5
C21—C20—C19	120.92 (13)	H33A—C33—H33C	109.5
C21—C20—H20	119.5	H33B—C33—H33C	109.5
C19—C20—H20	119.5	C32—C34—H34A	109.5
C20—C21—C22	121.07 (13)	C32—C34—H34B	109.5
C20—C21—H21	119.5	H34A—C34—H34B	109.5
C22—C21—H21	119.5	C32—C34—H34C	109.5
C23—C22—C21	117.51 (13)	H34A—C34—H34C	109.5
C23—C22—C31	121.81 (13)	H34B—C34—H34C	109.5
			
C25—O1—C2—C1	167.69 (14)	C9—C8—C12—O4	−59.53 (17)
C25—O1—C2—C3	−16.9 (2)	C7—C8—C12—C19	−71.74 (16)
C9—C1—C2—O1	174.96 (12)	C9—C8—C12—C19	120.94 (14)
C11—C1—C2—O1	4.7 (2)	O3—C11—C13—C18	−5.3 (2)
C9—C1—C2—C3	−0.6 (2)	C1—C11—C13—C18	174.38 (13)
C11—C1—C2—C3	−170.86 (13)	O3—C11—C13—C14	172.88 (13)
O1—C2—C3—C4	−172.57 (14)	C1—C11—C13—C14	−7.42 (19)
C1—C2—C3—C4	2.6 (2)	C18—C13—C14—C15	−0.3 (2)
C2—C3—C4—C10	−1.9 (2)	C11—C13—C14—C15	−178.56 (12)
C10—C5—C6—C7	−1.1 (2)	C13—C14—C15—C16	−0.2 (2)
C26—O2—C7—C8	−179.50 (13)	C14—C15—C16—C17	0.4 (2)
C26—O2—C7—C6	−2.6 (2)	C14—C15—C16—C27	−178.11 (15)
C5—C6—C7—O2	−174.82 (14)	C15—C16—C17—C18	−0.2 (3)
C5—C6—C7—C8	1.9 (2)	C27—C16—C17—C18	178.35 (16)
O2—C7—C8—C9	176.77 (12)	C16—C17—C18—C13	−0.4 (3)
C6—C7—C8—C9	−0.2 (2)	C14—C13—C18—C17	0.6 (2)
O2—C7—C8—C12	9.30 (19)	C11—C13—C18—C17	178.87 (14)
C6—C7—C8—C12	−167.63 (13)	O4—C12—C19—C24	2.49 (18)
C2—C1—C9—C10	−2.1 (2)	C8—C12—C19—C24	−178.00 (12)
C11—C1—C9—C10	167.73 (12)	O4—C12—C19—C20	−178.28 (12)
C2—C1—C9—C8	178.13 (13)	C8—C12—C19—C20	1.24 (18)
C11—C1—C9—C8	−12.1 (2)	C24—C19—C20—C21	−1.77 (19)
C7—C8—C9—C10	−2.21 (19)	C12—C19—C20—C21	178.99 (12)
C12—C8—C9—C10	164.91 (12)	C19—C20—C21—C22	−0.1 (2)
C7—C8—C9—C1	177.60 (13)	C20—C21—C22—C23	1.6 (2)
C12—C8—C9—C1	−15.3 (2)	C20—C21—C22—C31	−175.83 (12)
C6—C5—C10—C4	179.11 (14)	C21—C22—C23—C24	−1.3 (2)
C6—C5—C10—C9	−1.3 (2)	C31—C22—C23—C24	176.11 (13)
C3—C4—C10—C5	178.87 (14)	C22—C23—C24—C19	−0.5 (2)
C3—C4—C10—C9	−0.7 (2)	C20—C19—C24—C23	2.1 (2)
C1—C9—C10—C5	−176.89 (12)	C12—C19—C24—C23	−178.65 (12)
C8—C9—C10—C5	2.9 (2)	C17—C16—C27—C28	−108.6 (2)
C1—C9—C10—C4	2.7 (2)	C15—C16—C27—C28	69.9 (2)
C8—C9—C10—C4	−177.46 (12)	C16—C27—C28—C30	56.9 (2)
C2—C1—C11—O3	111.13 (15)	C16—C27—C28—C29	−178.08 (17)
C9—C1—C11—O3	−58.87 (18)	C23—C22—C31—C32	−108.55 (16)
C2—C1—C11—C13	−68.58 (17)	C21—C22—C31—C32	68.78 (18)
C9—C1—C11—C13	121.42 (15)	C22—C31—C32—C33	52.40 (18)
C7—C8—C12—O4	107.79 (15)	C22—C31—C32—C34	175.80 (14)

### Hydrogen-bond geometry (Å, º)

**Table:**

*D*—H···*A*	*D*—H	H···*A*	*D*···*A*	*D*—H···*A*
C21—H21···O4^i^	0.95	2.34	3.2716 (18)	167

Symmetry code: (i) *x*, *y*−1, *z*.
